# Dysphagia Lusoria Because of Congenital Double Aortic Arch

**DOI:** 10.14309/crj.0000000000000809

**Published:** 2022-06-23

**Authors:** Michael P. Meyers, Christopher John Murphy

**Affiliations:** 1 Mercy Health St Mary's Hospital, Grand Rapids, MI

## CASE REPORT

A 61-year-old woman presented with a 5-year history of intermittent, nonprogressive solid food dysphagia. She endorsed mild, occasional heartburn and reflux but had no other red flag symptoms. An esophagogastroduodenoscopy was performed, which identified pulsating, moderate extrinsic compression in the upper third of the esophagus (Figue 1). A chest computed tomography scan angiography revealed a complete vascular ring, composed of a double aortic arch, encasing both the esophagus and trachea (Figures 2, 3). A more complete history revealed several episodes of pneumonia since the onset of her dysphagia. A bronchoscopy confirmed 30%–40% tracheal obstruction for a length of 2 tracheal rings. Conservative management led to improvement in her symptoms.

**Figure 1.**
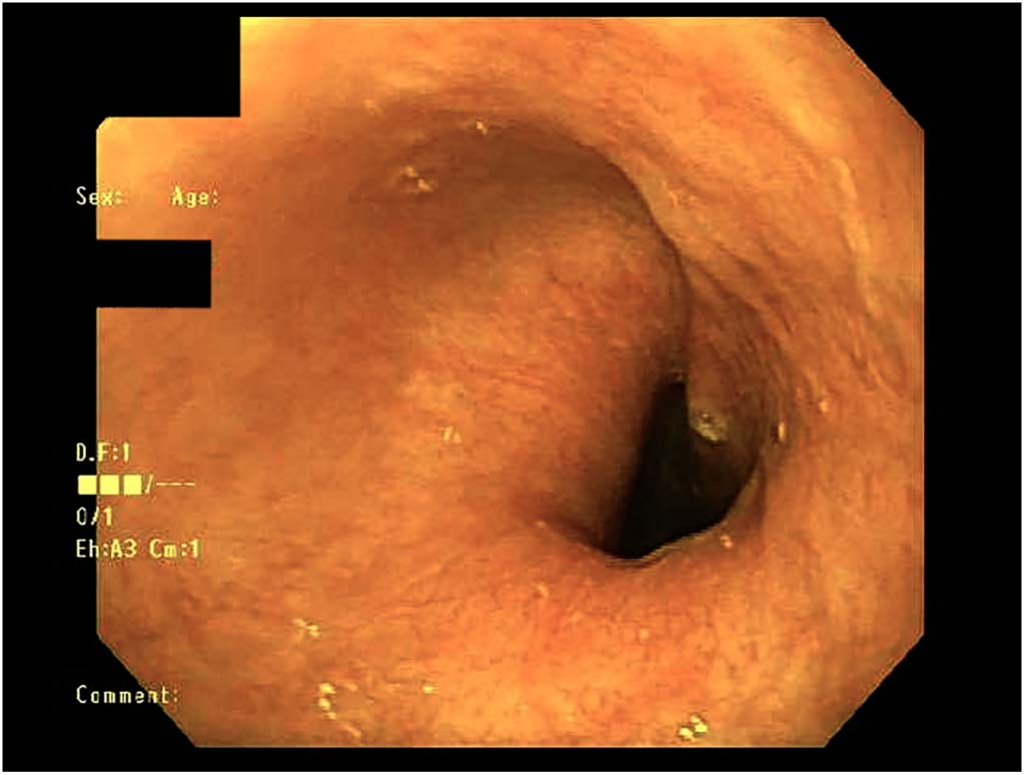
Endoscopic findings of extrinsic compression of the upper esophagus.

**Figure 2.**
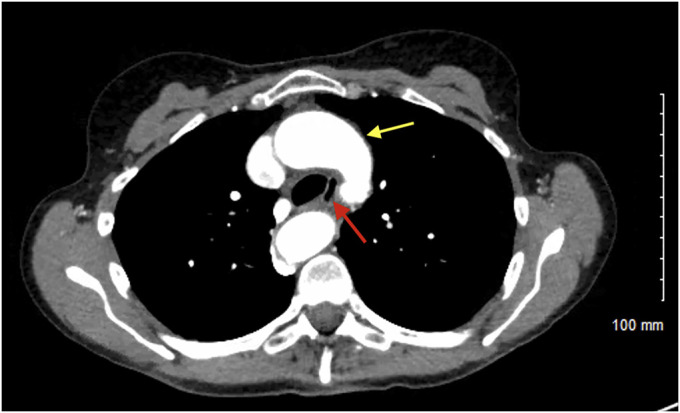
Chest computed tomography angiography demonstrating vascular ring from a double aortic arch (yellow arrow) encasing the trachea and esophagus (red arrow).

**Figure 3.**
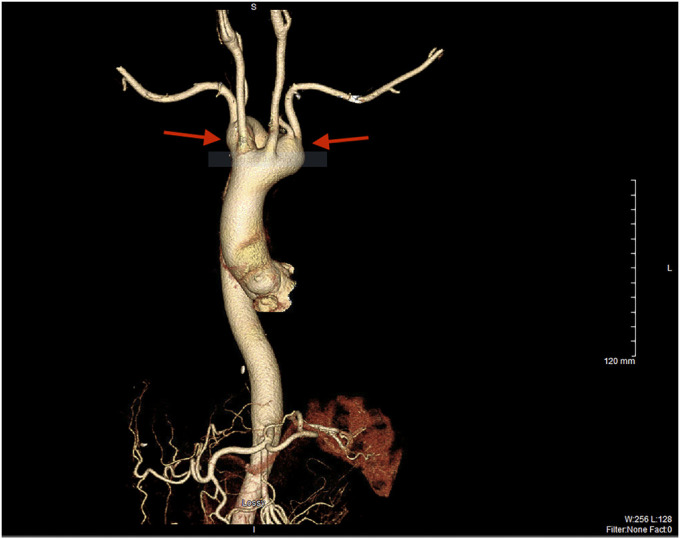
3-D vascular reconstruction highlighting the congenital double aortic arch (red arrows).

Extrinsic compression of the esophagus from aberrant vascular anatomy is a rare cause of dysphagia that was first described in 1794 by Dr David Bayford as “dysphagia lusoria,” which translates to “dysphagia by freak of nature.”^1^ Vascular rings encase both the trachea and esophagus, leading to potential symptoms of dyspnea on exertion, recurrent pneumonia, bronchitis, stridor, and dysphagia.^2^ Vascular rings are most commonly identified in pediatric populations. Late onset of dysphagia, as in our patient, has been attributed to progressive vascular pathology and age-related impairment of esophageal motility.^3^

Dysphagia lusoria will demonstrate extrinsic compression of the upper esophagus on esophagram or upper endoscopy. The diagnostic test of choice is computed tomography or magnetic resonance angiography. Mild symptoms can be managed with dietary adjustments. If conservative therapy fails, treatment is surgical repair.^4^ This case highlights that gastroenterologists must maintain clinical suspicion for vascular rings as a rare cause of dysphagia.

## DISCLOSURES

Author contributions: CJ Murphy is the article guarantor. All authors contributed equally to this manuscript.

Financial disclosure: None to report.

Informed consent was obtained for this case report.
